# The Effects of Stress at Work and at Home on Inflammation and Endothelial Dysfunction

**DOI:** 10.1371/journal.pone.0094474

**Published:** 2014-04-10

**Authors:** Amy L. Non, Eric B. Rimm, Ichiro Kawachi, Marissa A. Rewak, Laura D. Kubzansky

**Affiliations:** 1 Department of Anthropology, Vanderbilt University, Nashville, Tennessee, United States of America; 2 Departments of Epidemiology and Nutrition, Harvard School of Public Health, Boston, Massachusetts, United States of America; 3 Department of Social and Behavioral Sciences, Harvard School of Public Health, Boston, Massachusetts, United States of America; Idaho State University, United States of America

## Abstract

This study examined whether stress at work and at home may be related to dysregulation of inflammation and endothelial function, two important contributors to the development of cardiovascular disease. In order to explore potential biological mechanisms linking stress with cardiovascular health, we investigated cross-sectional associations between stress at work and at home with an inflammation score (n's range from 406–433) and with two endothelial biomarkers (intercellular and vascular adhesion molecules, sICAM-1 and sVCAM-1; n's range from 205–235) in a cohort of healthy US male health professionals. No associations were found between stress at work or at home and inflammation. Men with high or medium levels of stress at work had significantly higher levels of sVCAM-1 (13% increase) and marginally higher levels of sICAM-1 (9% increase), relative to those reporting low stress at work, independent of health behaviors. Men with high levels of stress at home had marginally higher levels of both sVCAM-1 and sICAM-1 than those with low stress at home. While lack of findings related to inflammation are somewhat surprising, if replicated in future studies, these findings may suggest that endothelial dysfunction is an important biological mechanism linking stress at work with cardiovascular health outcomes in men.

## Introduction

A large literature supports the relationship between chronic psychosocial stress and various cardiometabolic outcomes including cardiovascular disease (CVD) [1] and metabolic syndrome [2]. While psychosocial stress likely affects cardiometabolic health in part, by altering health behaviors such as increased cigarette smoking or poor diet, the association is often maintained even after accounting for many health behaviors [1]. A growing body of work suggests that direct biological mechanisms may also be important.

Systemic inflammation has been considered a likely pathway linking chronic psychosocial stress with many cardiovascular outcomes [3]. Stress may induce inflammation by triggering a release of cortisol and catecholamines that can initiate an inflammatory response via production of cytokines and acute phase reactants [4]. While an inflammatory response to acute stress can be adaptive, a chronic state of inflammation may develop in the context of ongoing psychosocial stress, which can lead to atherosclerotic processes [4]. Some studies have demonstrated links between psychosocial stressors and inflammation, most often measured with C-reactive Protein (CRP) and interleukin 6 (IL-6) [5]. However, these associations are not always consistent; e.g. some researchers have found CRP to be only marginally associated with perceived stress, and not at all associated with chronic stress, social support, or loneliness [6].

Endothelial dysfunction has recently emerged as a related and potentially important pathway through which psychosocial stress may influence cardiometabolic health, as it is associated with insulin resistance and may be causally related to early atherosclerotic CVD and type II diabetes [7]. One common measure of endothelial dysfunction is flow mediated dilation (FMD), which is a measure of how arteries dilate in response to reactive hyperemia, with greater dilation indicating better endothelial function. One study has demonstrated a significant negative association between caregiving stress and FMD [8]. FMD however, is less commonly measured in large scale population-based studies, limiting prior work examining endothelial function and stress. Two less well-studied markers of endothelial function include soluble intercellular adhesion molecule (sICAM-1) and soluble vascular cellular adhesion molecule (sVCAM-1). Levels of these molecules are upregulated on the surface of vascular endothelial cells in response to stress-induced activation of proinflammatory cytokines [7]. Both sICAM-1 and sVCAM-1 mediate transendothelial migration of leukocytes, which can lead to vascular inflammation and atherosclerosis [9]. In concert with findings on FMD, this work suggest that changes in endothelial function may lie on the pathway linking stressful experiences with CVD, and that sICAM-I and sVCAM-I may be useful for tracking these processes.

Prior work has indicated that job strain (defined as high job demands, low job control) is sometimes, but not always, associated with inflammatory markers [10]. Four out of five studies to date did not find evidence for an association between job strain and inflammation [11], [12], though one study found that higher job demands, and independently, lower social support at work, both demonstrated increased CRP levels [13]. Fewer studies have examined job stress and endothelial biomarkers. One study found rotating shift workers exhibited higher job strain and reduced endothelial function (measured by fingertip peripheral arterial tonometry), relative to daytime only workers [14]. Hardly any work has considered whether stress at home, particularly among men, might have similarly disruptive effects on these biological processes. Thus, whether effects are unique to work-related stress or a function of stress more generally has not been explicitly considered.

We investigate the cross-sectional associations between self-reported stress at home and at work with an inflammation score derived from measures of CRP, IL-6, and tumor necrosis factor-α receptors (sTNFR-1, and sTNFR-2), and with 2 biomarkers of endothelial function, sICAM-1 and sVCAM-1, in a subset of a large cohort of US male health professionals. Inflammatory markers in the inflammation score were selected based on prior work demonstrating their association with acute stress under laboratory conditions, or with early life adversity [15], [16]. We hypothesize that men who report higher stress levels at work or at home will a have higher level of inflammation and higher concentrations of sICAM-1 and sVCAM-1 than men who report lower stress at work or at home. We also investigate if potential pathway variables, such as health behaviors, alter or help to explain the associations of interest.

## Methods

### Ethics Statement

The study was approved by the IRB at the Harvard University School of Public Health, and responses to the questionnaires constitute written informed consent.

Participants are from a subset of the Health Professional Follow-Up Study (HPFS), an ongoing cohort study of men's health established in 1986. HPFS began with detailed diet and medical history questionnaires from US male health professionals between ages 40 and 75 years at study initiation, with follow-up questionnaires every 2 years. In 1992, measures of stress at work and at home were included in the questionnaire, so this served as the baseline for the current study. Measures of inflammation and endothelial function were variously available from two subsequent sub-studies, a nested case-control study of coronary heart disease (CHD) that included 532 men without CHD [17], and 422 men from a study of alcohol and heart disease who were not also in the nested case-control study [18].

For analyses with inflammation, we included the men without CHD from the nested case-control study of CHD and then added the additional 442 men from the study on alcohol and heart disease yielding a total of 954 men who had complete data on all inflammatory markers. As men with chronic conditions may also have other health problems that make it more difficult to detect associations of interest, we excluded any men with other chronic health conditions, such as high blood pressure (BP), high cholesterol, or diabetes. Thus, after removing men with outlying scores on any of the inflammatory markers (n = 68), and men with a history of these chronic health conditions as of 1992 (n = 408), our final sample for these analyses included 406 healthy men with reported stress at work, and 443 healthy men with reported stress at home.

Measures of endothelial function were available only among the 532 men free of CHD from the nested case-control study of CHD. From this subset, we excluded 281 men with a history of high BP, high cholesterol or diabetes as of 1992. We also excluded six men with outlying values for sICAM-1 and sVCAM-1, based on a generalized extreme studentized many-outlier detection method [19]. This resulted in a final study sample for analyses with endothelial function of 205 men who reported stress levels at work and 235 men who reported stress levels at home.

### Stress measures

A 2-part question on general stress experienced either at home or at work was asked in the 1992 questionnaire: “How would you rate the amount of stress in your daily life a) at work b) at home?” Response options included severe, moderate, light, and minimal, and these were rescored as high (severe or moderate), medium (light), and low (minimal) stress categories to account for low numbers in some categories, and also to maintain our ability to assess whether there might be a dose-response relation with increasing levels of stress. Although the medium category may seem somewhat less robust, prior work has suggested that even small increases in psychological factors may be associated with monotonic increases in risk or levels of health-related outcomes [20]. While these are single item measures, they are face valid and use of such measures is not an uncommon practice in epidemiology, where researchers must trade off the opportunity to look at these questions with somewhat limited measures [21]. Stress at home and at work were analyzed separately and treated as categorical variables in main analyses, and as continuous variables based on scores assigned to the 3 derived categories (low  = 0, medium  = 1, high  = 2) in sensitivity analyses. The 2 stress items were also combined to create a combined measure of total stress for men, also classified as low, medium, and high).

### Measurement of biomarkers

Blood was drawn by local phlebotomists and returned by mail on ice within 24 hours for 95% of samples. Whole blood was separated into plasma, buffy coat, and red blood cells by centrifugation and stored in liquid nitrogen. Details about the measurement of sICAM-1, sVCAM-1, IL-6, and sTNFRs have been previously described [22]. In brief, these biomarkers were measured by ELISA (R&D Systems, Minneapolis, MN, USA) on a Hitachi 911 analyzer (Roche Diagnostics, Indianapolis, IN, USA), with coefficient of variation (CV) ranging from 8.7 to 9.3%_ENREF_15. CRP was measured using a highly sensitive immunoturbidimetric assay (Denka Seiken, Niigata, Japan), with a CV <6% [22].

### Covariates

Information on men's age (years) and self-reported race (White, Black, Asian, or other) was collected at baseline (1986). Smoking habits (current, past, never), total physical activity level (quintiles of metabolic equivalent/week), body mass index (BMI, kg/m^2^, treated as a continuous variable). Food intake was summarized by an alternative healthy eating index (aHEI, grouped into quintiles) according to aspects of diet measured in 1990. The aHEI is a validated measure of diet quality based on modified recommendations from the U.S. Department of Agriculture [23]. A missing category was modeled for discrete variables of smoking (n = 13 missing), aHEI (n = 12 missing), and activity level (n = 3 missing).

### Statistical analyses

Statistical analyses were conducted using SAS version 9.2 (SAS Institute, Cary, North Carolina). When considering the inflammatory markers, an inflammation score was created by summing the number of inflammatory markers for which the participant scored in the high risk category (top quartile, or above diagnostic cutoff of 0.3 mg/dl for CRP), following similar work [24]. In prior studies, stronger associations of health outcomes with a summary measure versus individual markers have been found [25]. In the present study, scores ranged from 0-3 with high scores indicating higher levels of inflammation. Primary analyses used this additive index of the four inflammatory markers to provide insight into overall systemic imbalance. However, we also considered each (log-transformed) inflammatory marker as a separate outcome. When considering the markers of endothelial function, each marker was considered separately.

We tested for primary associations using multiple linear regression. For each outcome, two models were evaluated. The first model included demographic covariates of age and self-identified race, with all non-White races collapsed into one category due to small sample size. A second ‘pathway’ model added a set of health behavior variables that may be on the pathway between stress and alterations in inflammatory processes or endothelial function (e.g. smoking, diet). Percent increase in the outcome associated with higher versus lower stress was calculated by dividing the estimate of the adjusted least square mean predicted value for the outcome in the highest stress category by the predicted value in the lowest stress category and multiplying by 100. All analyses were repeated using robust variance (Proc Mixed in SAS v9.2), to ensure validity without requiring normal distribution assumptions._ENREF_20 Poisson models were also used for analyses with the inflammation score, and multivariable linear regression was used when considering each inflammatory marker individually as a log-transformed continuous outcome.

## Results

Characteristics of the study population are presented according to stress level at work and at home (Table 1). Men with the least stress at work were significantly older and had higher physical activity; men with the least stress at home were also significantly older, had higher physical activity, were marginally less likely to be White, and to have lower BMI. Those missing data on stress at work differed from those not missing data in that they were significantly older and had lower BMI, while those missing data on stress at home were significantly older.

**Table 1:** Distribution of population characteristics by stress level at work and at home.

	Stress at Work (n = 406)				Stress at Home (n = 443)			
	Low	Medium	High	P-Value^a^	Low	Medium	High	P-Value^a^
	*n* = 35	*n = *101	*n* = 270		*n* = 150	*n = *192	*n* = 101	
**Demographics**								
Mean age (SD), years	65.4 (7.8)	60.2 (8.9)	57.7 (8.3)	**<0.001**	61.6 (8.8)	59.3 (8.9)	58.2 (8.6)	**<0.007**
Race, n (%)								
White	29 (82.9)	94 (93.1)	248 (91.9)	0.160	130 (86.7)	181 (94.3)	93 (92.1)	**0.045**
Non-White/Other	6 (17.1)	7 (6.9)	22 (8.2)		20 (13.3)	11 (5.7)	8 (7.9)	
**Health Behaviors**								
Smoking, n (%)								
Current	3 (8.6)	8 (7.9)	29 (10.7)	0.066	11 (7.3)	18 (9.4)	13 (12.9)	0.369
Past	14 (40.0)	36 (35.6)	133 (49.3)		68 (45.3)	87 (45.3)	49 (48.5)	
Never	15 (42.9)	53 (52.5)	102 (37.8)		63 (42.0)	82 (42.7)	38 (37.6)	
Mean Physical Activity (SD), mets/week	61.9 (53.9)	39.2 (31.9)	40.7 (47.1)	**0.022**	49.3 (48.6)	38.5 (42.8)	38.1 (36.4)	**0.045**
Mean BMI (SD), kg/m2	25.2 (2.8)	24.6 (2.5)	25.3 (3.0)	0.094	24.7 (2.7)	25.0 (2.7)	25.6 (3.4)	**0.049**
Mean aHEI score (SD)	46.7 (11.7)	46.0 (10.6)	44.8 (11.2)	0.475	45.7 (11.2)	45.7 (11.5)	44.2 (10.1)	0.505

a P-value from ANOVA for continuous variables, from Chi Square for categorical variables (P-values significant at alpha = 0.05 shown in bold). Distribution of characteristics similar to smaller sample of men with data on sICAM-1/sVCAM-1. Abbreviations: aHEI, alternative healthy eating index; BMI, body mass index; mets, metabolic equivalents; SD, standard deviation.

### Stress and inflammation

Associations of stress at work and at home with the inflammation score were uniformly positive, but none reached statistical significance (Table 2). These findings remained unchanged after adjusting for health behaviors. Poisson models showed the same substantive pattern as linear models. When each inflammatory marker was tested as a separate outcome, none showed statistically significant associations with stress at work or at home (Table S1).

**Table 2:** Linear Regression models for stress at work and at home and inflammation, sVCAM-1, and sICAM-1 among healthy men.

	Inflammation Score		sVCAM-1 (ng/ml)		sICAM-1 (ng/ml)	
	B (SE)		B (SE)		B (SE)	
	P-Value		P-Value		P-Value	
Stress at work	Model A^a^	Model B^b^	Model A^a^	Model B^b^	Model A^a^	Model B^b^
	(n = 406)	(n = 406)	(n = 205)	(n = 205)	(n = 205)	(n = 205)
High vs Low	0.06 (0.17)	0.10 (0.17)	**144.78 (62.13)**	**134.10 (60.92)**	26.78 (16.60)	26.68 (16.00)
	0.718	0.571	**0.021**	**0.028**	0.109	0.097
Medium vs Low	0.07 (0.18)	0.13 (0.18)	**146.00 (66.50)**	**144.10 (64.26)**	23.92 (17.78)	24.63 (16.88)
	0.694	0.460	**0.029**	**0.026**	0.180	0.146
Stress at home	Model A^b^	Model B^b^	Model A^a^	Model B^b^	Model A^a^	Model B^b^
	(n = 443)	(n = 443)	(n = 235)	(n = 235)	(n = 235)	(n = 235)
High vs Low	0.12 (0.12)	0.07 (0.12)	**88.90 (45.92)**	60.95 (46.30)	**23.85 (12.25)**	16.75 (11.87)
	0.329	0.553	**0.054**	0.190	**0.053**	0.160
Medium vs Low	0.02 (0.10)	0.00 (0.10)	38.90 (39.65)	44.38 (39.55)	13.47 (10.58)	9.93 (10.13)
	0.882	0.981	0.328	0.263	0.204	0.328

a Model A is adjusted for age and self-reported race.

b Model B is additionally adjusted for health behaviors (smoking, diet, exercise, and BMI-continuous).

Beta coefficients (SE) and P-values significant at alpha ≤0.05 shown in bold.

### Stress at work and sVCAM-1/sICAM-1

Men with high or medium stress at work had higher levels of sVCAM-1 relative to men with low stress at work (Table 2). Translating to a percent increase, in age- and race-adjusted regression models, men with high or medium stress at work each had 13% higher levels of sVCAM-1 relative to those with low work stress. After health behavior covariates were added to the models, no covariates were significantly associated with sVCAM-1, and the relationship between stress at work and sVCAM-1 was unchanged (Table 2). A marginally significant relationship (p≤0.1) was found between high stress at work and sICAM-1 (9% higher levels), and this relationship was unchanged by the addition of health behaviors (Table 2).

### Stress at home and sVCAM-1/sICAM-1

High stress at home was marginally associated (p<0.1) with both sICAM-1 and sVCAM-1 (Table 2). Specifically, after translating to a percent increase, men with the highest stress had 7.2% higher sVCAM-1 and 8.1% higher sICAM-1 than those with low stress at home (Table 2). Adding behaviors to these models attenuated these associations to non-significance, possibly suggesting that some of the effect of stress at home is carried by behavior-related factors. For example, greater stress at home was significantly associated with higher BMI and less physical activity (Table 1).

### Additional analyses

A combined measure summed across stress at home and at work was marginally associated (p<0.1) with sVCAM-1, but not with sICAM-1. When considering each stress as a continuous measure, stress at work was marginally associated with sVCAM-1 (b = 49.41, S.E. = 27.6, p = 0.08), but not with sICAM-1 (b = 10.10, S.E. = 7.35, p = 0.17), and stress at home was significantly associated with sVCAM-1 (b = 44.05, S.E. = 22.77, p = 0.05), and with sICAM-1 (b = 12.04, S.E. = 6.07, p = 0.05). Interactions between stress at home and stress at work were not significant in any models. In all analyses where robust variance was applied, results did not change.

## Discussion

Somewhat surprisingly, we identified no consistent association between stress at work or at home and inflammation. However a consistent association did emerge between stress at work and sVCAM-1 in male health professionals, independent of health behaviors. We also found evidence of marginal associations for stress at work with sICAM-1, and for stress at home with both endothelial markers. These findings suggest stress may be acting partly through direct dysregulation of endothelial function. Thus, these endothelial biomarkers may be important risk factors to pursue in further research.

The lack of association between stress and inflammation in this study was unexpected, as prior research has shown a link between chronic stress and elevated inflammation [5], [24]. That said, some prior work has also failed to find this association [11], [12], [26]. In the current study, lack of an association may be because these measures of stress were not associated with BMI in our sample of professional healthy men, unlike in other samples [27], and inflammation is highly sensitive to BMI. Given that other studies have also failed to find a consistent association, it is possible that the relationship is moderated by other as-yet unidentified factors. Further prospective studies are needed to explore this question with more detailed measures of stress and inflammation in more diverse populations.

Though little work has investigated associations between stress and adhesion molecules, some research has demonstrated associations between stress and flow-mediated dilation (FMD) [8]. Our work is consistent with these studies, but our focus on adhesion molecules allows us to pinpoint a more targeted cellular level-mechanism than does FMD alone. However, since our study is the first to investigate associations for stress at work and at home with adhesion molecules, further work is needed to assess whether findings hold across other cardiometabolic outcomes and in other populations. Few studies have directly compared the health effects of stress at work with stress at home in men. In the Whitehall II Study, a lack of control in the home and the work environment both increased risks of developing depression and anxiety for men, though effects differed according to social class [28]. While our study showed stronger associations with stress at work and VCAM-1, we also identified some associations between stress at home with both sICAM-1 and sVCAM-1.

The biological mechanisms linking stress with endothelial function are yet unknown. One potential pathway is through stress-induced activation of proinflammatory cytokines, which have shown to induce expression of adhesion molecules on the surface of vascular endothelial cells [7]. Though we did not find an increase in inflammatory cytokines directly, there may be increased inflammation in other unmeasured markers, such as interleukin-1β, which upregulates expression of both ICAM and VCAM [9]. Stress can also lead to chronic activation of the sympathetic nervous system, leading to hemodynamic changes such as increased blood pressure, which can injure the endothelium [8].

A prior study in this population has shown that a similar increase in sVCAM-1 (e.g. from lowest to second quintile) corresponds to a positive but non-significant relative risk for developing CHD of 1.31 (CI: 0.79, 2.18) [17]. A significantly higher risk of incident CHD (nearly 2.5 fold) was found when levels of both sICAM-1 and sVCAM-1 were elevated [17]. Epidemiological studies suggest different roles for these molecules in atherosclerosis, where sICAM-1 may be strongly associated with CHD risk in healthy populations [29] while sVCAM-1 is more strongly associated with disease progression [30].

Our study has several potential limitations. The relative homogeneity of this cohort limits generalizability of these results, though it also reduces concerns about residual confounding from unmeasured factors. As our study is limited to highly educated men, we may be underestimating effects, and might see even stronger associations in less advantaged populations. Single-item measures of stress at home and at work do not capture the many dimensions of stress experience. Only cross-sectional associations were considered, and we cannot rule out the possibility, though unlikely, that alterations in sVCAM-1 may influence psychosocial stress rather than vice versa. Additionally, it is possible that some unmeasured confounder may lead both to higher stress and endothelial dysfunction. Strengths of this study include a well-characterized sample, and limited concerns about self-report bias, as the outcomes were objectively measured.

In conclusion, associations between stress at home or stress at work and inflammation were not evident, but some associations with endothelial dysfunction were identified among healthy men. We identified relatively robust associations between stress at work and sVCAM-1 and somewhat more modest associations between stress at home with both sVCAM-1 and sICAM-1. Should they be replicated, these findings suggest that further work is warranted to identify whether a direct biological mechanism related to endothelial dysfunction may link stress at work with cardiovascular health in men. Monitoring endothelial function may provide insight into how these experiences alter functioning prior to disease development. Additional work might also consider whether there are factors that modify the association between psychological stress and inflammation. Ultimately, future studies might investigate if interventions or policies designed to reduce stress at work and at home may be beneficial in improving cardiovascular health and reducing risk of heart disease.

## Supporting Information

Table S1 **Linear Regression Models for stress at work and at home and individual inflammatory markers among healthy men.** ^a^Model A is adjusted for age and self-reported race. ^b^Model B is additionally adjusted for health behaviors (smoking, diet, exercise, and BMI-continuous). All inflammatory biomarkers were transformed by taking the natural logarithm.
(DOCX)
