# Supplementary material for: The Effects of Stress at Work and at Home on Inflammation and Endothelial Dysfunction
Source: PLoS One. 2014 Apr 10;9(4):e94474. doi: 10.1371/journal.pone.0094474 (PMC3983187; doi:10.1371/journal.pone.0094474)
Supplement: Table S1 — Linear Regression Models for stress at work and at home and individual inflammatory markers among healthy men. aModel A is adjusted for age and self-reported race. bModel B is additionally adjusted for health behaviors (smoking, diet, exercise, and BMI-continuous). All inflammatory biomarkers were transformed by taking the natural logarithm. (DOCX) [file pone.0094474.s001.docx]

Table S1. Linear Regression Models for stress at work and at home and individual inflammatory markers among healthy men

|  | CRP (mg/dl)  (n=571) | | IL-6 (pg/ml)  (n=406) | | | sTNFR-1 (pg/ml)  (n=406) | | | | | | sTNFR-2 (pg/ml)  (n=406) | | |  |
| --- | --- | --- | --- | --- | --- | --- | --- | --- | --- | --- | --- | --- | --- | --- | --- |
|  | Model A^a^ | Model B^b^ | Model A^a^ | Model B^b^ | | Model A^a^ | | Model B^b^ | | Model A^a^ | | | Model B^b^ | |  |
| Stress at work | B (SE)  P-value | B (SE)  P-value | B (SE)  P-value | B (SE)  P-value | | B (SE)  P-value | | B (SE)  P-value | | B (SE)  P-value | | | B (SE)  P-value | |  |
| High vs Low | 0.24 (0.22)  0.277 | 0.25 (0.21)  0.236 | 0.09 (0.13)  0.457 | 0.09 (0.13)  0.479 | | 0.04 (0.05)  0.435 | | 0.05 (0.05)  0.289 | | 0.00 (0.07)  0.982 | | | 0.00 (0.07)  0.900 | |  |
| Med vs low | -0.25 (0.23)  0.297 | -0.19 (0.23)  0.416 | -0.05 (0.14)  0.714 | -0.01 (0.14)  0.927 | | 0.06 (0.05)  0.294 | | 0.07 (0.06)  0.195 | | 0.05 (0.08)  0.535 | | | 0.06 (0.07)  0.417 | |  |
|  | CRP (mg/dl)  (n=620) | | IL-6 (pg/ml)  (n=443) | | | sTNFR-1 (pg/ml)  (n=443) | | | | sTNFR-2 (pg/ml)  (n=443) | | | | |  |
| Stress at home | Model A^a^  B (SE)  P-value | Model B^b^  B (SE)  P-value | Model A^a^  B (SE)  P-value | Model B^b^  B (SE)  P-value | | Model A^a^  B (SE)  P-value | | Model B^b^  B (SE)  P-value | | Model A^a^  B (SE)  P-value | | | Model B^b^  B (SE)  P-value | |  |
| High vs low | 0.06 (0.16)  0.682 | 0.00 (0.15)  0.958 | -0.03 (0.10)  0.712 | | -0.10 (0.09)  0.249 | | -0.02 (0.04)  0.610 | | -0.03 (0.04)  0.474 | | -0.02 (0.05)  0.744 | | | -0.02 (0.05)  0.647 | |
| Med vs low | 0.07 (0.13)  0.620 | 0.07 (0.13)  0.594 | 0.01 (0.08)  0.920 | | -0.02 (0.08)  0.807 | | -0.01 (0.03)  0.780 | | 0.00 (0.03)  0.821 | | -0.02 (0.04)  0.629 | | | -0.02 (0.04)  0.585 | |

^a^Model A is adjusted for age and self-reported race. ^b^Model B is additionally adjusted for health behaviors (smoking, diet, exercise, and BMI-continuous). All inflammatory biomarkers were transformed by taking the natural logarithm.
